# Intermediate Cluster Disinfection: Which Disinfection Solution Is Most Effective on Milking Liners? A Comparison of Microorganism Reduction on Liner Inner Surfaces Using Quantitative Swab Sampling Technique

**DOI:** 10.3390/pathogens12040560

**Published:** 2023-04-06

**Authors:** Sabrina Scheib, Stefanie Leimbach, Georg Avramidis, Martin Bellmann, Julia Nitz, Christian Ochs, Anne Tellen, Nicole Wente, Yanchao Zhang, Wolfgang Viöl, Volker Krömker

**Affiliations:** 1 Department of Microbiology, Faculty of Mechanical and Bioprocess Engineering, University of Applied Sciences and Arts, 30452 Hannover, Germany; sabrina.scheib@hs-hannover.de (S.S.); stefanie.leimbach@hs-hannover.de (S.L.); julia.nitz@hs-hannover.de (J.N.); anne.schmenger@t-online.de (A.T.); nicole.wente@hs-hannover.de (N.W.); yanchao.zhang@hs-hannover.de (Y.Z.); 2 Faculty Engineering and Health, University of Applied Sciences and Arts, 37085 Goettingen, Germany; georg.avramidis@hawk.de (G.A.); martin.bellmann@hawk.de (M.B.); christian.ochs1@hawk.de (C.O.); wolfgang.vioel@hawk.de (W.V.); 3 Department of Veterinary and Animal Sciences, University of Copenhagen, 1870 Frederiksberg C, Denmark

**Keywords:** Plasma-Activated Buffered Solution, Peracetic Acid Solution, wet–dry swab technique, mastitis prevention, surface microbial count

## Abstract

During machine milking, pathogenic microorganisms can be transmitted from cow to cow through liners. Therefore, in Germany, a spray method for the intermediate disinfection of the milking cluster is often used for prevention. This method of cluster disinfection is easy to perform, requires little time and no extra materials, and the disinfection solution is safe from outside contamination in the spray bottle. Since no data on a systematic efficacy trial are available, the aim of this study was to determine the microbial reduction effect of intermediate disinfection. Therefore, laboratory and field trials were conducted. In both trials, two sprays of 0.85 mL per burst of different disinfectant solutions were sprayed into the contaminated liners. For sampling, a quantitative swabbing method using a modified wet–dry swab (WDS) technique based on DIN 10113-1: 1997-07 was applied. Thus, the effectiveness of disinfectants based on Peracetic Acid, Hydrogen Peroxide and Plasma-Activated Buffered Solution (PABS) was compared. In the laboratory trial, the inner surfaces of liners were contaminated with pure cultures of *Escherichia* (*E.*) *coli*, *Staphylococcus* (*S.*) *aureus*, *Streptococcus* (*Sc.*) *uberis* and *Sc. agalactiae*. The disinfection of the contaminated liners with the disinfectants resulted in a significant reduction in bacteria with values averaging 1 log for *E. coli*, 0.7 log for *S. aureus,* 0.7 log for *Sc. uberis* and 0.8 log for *Sc. agalactiae*. The highest reduction was obtained for contamination with *E. coli* (1.3 log) and *Sc. uberis* (0.8 log) when PABS was applied and for contamination with *S. aureus* (1.1 log) and *Sc. agalactiae* (1 log) when Peracetic Acid Solution (PAS) was used. Treatment with sterile water only led to an average reduction of 0.4 log. In the field trial, after the milking of 575 cows, the liners were disinfected and the total microorganism count from the liner surface was performed. The reduction was measured against an untreated liner within the cluster. Although a reduction in microorganisms was achieved in the field trial, it was not significant. When using PAS, a log reduction of 0.3 was achieved; when using PABS, a log reduction of 0.2 was obtained. The difference between the two disinfection methods was also not significant. Treatment with sterile water only led to a reduction of 0.1 log. The results show that spray disinfection under these circumstances does result in a reduction in the bacteria on the milking liner surface, but for effective disinfection a higher reduction would be preferred.

## 1. Introduction

Mastitis is considered to be the most important disease in the dairy cattle sector from an economic point of view [1]. Due to the multifactorial nature of mastitis, it is necessary to prevent not only the transmission of pathogenic microorganisms from the environment, e.g., through the good cleanliness of the barn [2,3,4], but also the transmission of pathogens from cow to cow or quarter to quarter during milking. One possibility for the transmission of mastitis pathogens is the contamination of the teat skin and teat canal during milking [5,6]. In particular, cow-associated microorganisms are pathogens that are transmitted during milking via teat liners [7,8,9]. However, teat liners have also been shown in the past to be contaminated with environmental pathogens, such as *Sc. uberis*, after the milking of infected cows [10,11]. Thus, regardless of the pathogen group and species, the contamination of the liner, which is the direct connection between the udder and the milking machine, is possible [12].

As a preventive measure against the spread of mastitis pathogens during the milking process, the intermediate disinfection of the milking cluster is used and recommended in problem herds in Germany [12], as it is a way to prevent pathogen transmission [13,14]. On farms using automatic milking systems, without classical milking order as in conventionally milked herds and with considerably more cows using a milking cluster throughout the day, the intermediate disinfection of the milking clusters is essential [12,15,16].

Various methods are available for intermediate disinfection, such as dipping the cluster into a bucket, spraying the liners and various other commercially available disinfection systems such as backflush systems [17,18]. Köster et al. [19] showed that, out of 96 herds in Brandenburg, Germany, 65.1% of farms performed cluster disinfection after each cow and 3.8% of farms disinfectedthe cluster after the milking of mastitis cows. Different disinfection methods were used, the most common being disinfection performed by dipping the cluster inside of a bucket with a disinfectant. In most cases, a 0.1% Peracetic Acid Solution (PAS) was used as the solution for intermediate disinfection, when disinfection was carried out by hand. Preparations containing PAS show a good bactericidal effect, have a broad spectrum of activity and are effective over a wide temperature range [20,21,22]. Due to its rapid action and decomposition into acetic acid, water and oxygen, it is considered harmless from the point of view of residue hygiene, so that no withdrawal time results from its use [23,24]. In the case of a herd problem with cow-associated pathogens, such as *S. aureus*, for example, intermediate disinfection with PAS could lead to success [25]. Other disinfection solutions available on the market are ready-to-use mixtures containing hydrogen peroxide, peracetic acid and acetic acid, which are used for the standard disinfection of milking clusters, milking robot brushes and udder cloths. Like PAS, these must be diluted with water to an appropriate concentration before use. In an undiluted state, they can be irritating to the respiratory tract as well as to the eyes and skin [20,22].

As an environmentally friendly, non-thermal and non-toxic alternative to the conventional disinfectants, Plasma-Activated Liquids (PALs), such as Plasma-Activated Water and Plasma-Activated Buffered Solution (PABS), are described by many authors and increasingly being tested in the food industry [26,27,28]. Reactive oxygen and nitrogen species (RONS) are generated by the reaction of water or buffered solutions during plasma treatment, resulting in high biochemical activity. That is why they are used in biomedicine for various applications, for example, to remove biofilms, heal wounds, treat cancer and also inactivate bacteria [26,29]. For the wide use of PALs, especially in the inactivation of microorganisms, the transfer and solvation of RONS from the gas phase to the liquid phase is essential [30]. To these RONS belong long-lived species, such as nitrates, nitrites, hydrogen peroxide and ozone, but also short-lived radicals, such as hydroxyl radicals or atomic oxygen [26,31]. Thus, despite their fluctuating composition, the PALs produced can be used for several days depending on the storage temperature due to their remaining antimicrobial effect [32]. Even though it is already known that plasma-induced oxidative stress causes damage to the cell wall, proteins and DNA of bacteria [33,34], it is necessary to understand more precisely which RONS are involved crucially in the antimicrobial effect of PALs to improve the disinfection process [30].

Using the various intermediate disinfection methods, it is assumed that bacterial reduction will be achieved on the surface of the liners [5,18,35,36]. In former studies, it has already been shown, by means of swab samples, that pathogenic microorganisms could no longer be detected on the liner surface after disinfection [25,37]. However, the exact reduction in the surface microbial count on the liner is unknown, as a quantitative method is needed to determine it, which is mostly not used in practice. Therefore, in a previous study, a swab method was defined that is suitable for the quantitative detection of the surface bacterial count on the liner, using a modified wet–dry swab (WDS) technique in accordance to DIN 10113-1: 1997-07 [38]. This quantitative swab method on the liner can be used to measure the actual level of liner contamination and the reduction in the surface bacterial count due to disinfection with the various disinfectants available. For this purpose, both a laboratory trial and a field trial were carried out in this study to compare the microorganism reduction on liner inner surfaces after using different disinfection solutions using a quantitative swab sampling technique.

## 2. Materials and Methods

### 2.1. Laboratory Trial

**Figure 1:**
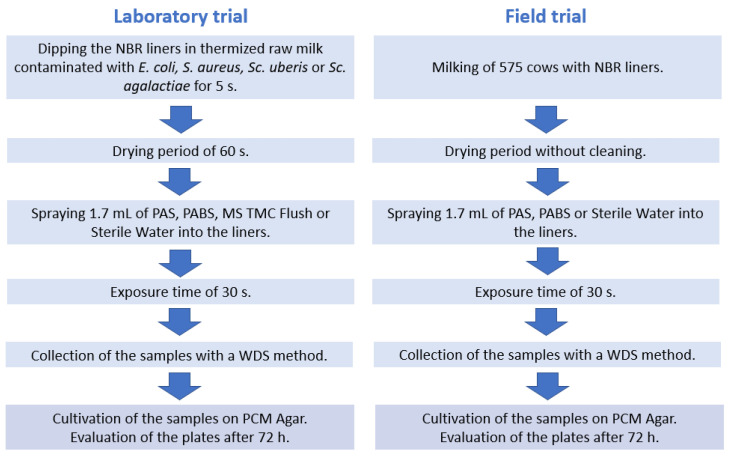
Diagram of the experimental setup. PAS: Peracetic Acid Solution, PABS: Plasma-Activated Buffered Solution, WDS: wet–dry swab.

In the laboratory trial, liners were contaminated with four different mastitis pathogens, by dipping them into contaminated milk for 5 s. After contamination and subsequent disinfection, the liners were sampled. For the contamination of the liners in the laboratory study, pathogen isolates of *E. coli* (DSMZ 1300), *S. aureus* (ATCC 700407), *Sc. uberis* (ATCC 12600) and *Sc. agalactiae* (wild isolate, Hanover University of Applied Sciences and Arts, St. No. 11881) were used. They were stored at −80 °C with the addition of glycerol until assayed, as is common practice for the long-term storage of bacteria [39]. The preculturing of each pathogen from the strain was performed for 24 h at 37 °C in brain–heart broth (Carl Roth GmbH & Co. KG, Karlsruhe, Germany). For the main culture, 10 µL of the preculture was incubated for 24 h at 37 °C in brain–heart broth again. Then, 40 µL of the different main cultures were each mixed with 399.96 mL of raw milk that had previously been thermized in a water bath at 60 °C for 50 min. Thus, a contaminated milk with a bacterial density of 10^4^ colony-forming units/mL (cfu/mL) was prepared and enumerated by the plate count method (Plate Count Agar with Powdered Milk, Carl Roth GmbH & Co. KG, Karlsruhe, Germany). These bacterial concentrations in the inoculated milk are representative of concentrations that can be found in intramammary infections [40], but they do not definitely indicate the level of bacteria that adhere to the liner surface. Autoclaved liners out of nitrile butadiene rubber (NBR) (WS029U, Milkrite, Aulendorf, Germany), which had been used for 2000 times over a 7-month period on one farm, were immersed upside down to a depth of 9 cm in the contaminated milk for 5 s. After inoculation, the liners were turned upside down for 60 s to dry. Subsequently, the inner surfaces of the liners were disinfected with either PAS (APPLICHEM GmbH, Darmstadt, Germany) at a concentration of 0.1%, PABS or the ready-to-use mixture of hydrogen peroxide, peracetic acid and acetic acid (MS TMC Flush, Schippers Europe B.V, Kerken, Germany) at a concentration of 1.25% or sterile water. For disinfection, two sprays of 0.85 mL each of the solutions at room temperature were applied into the openings of the liners with spray bottles. After an exposure time of 30 s, the samples were collected by swabbing as described in the previous study [38] (see Figure 1). For the positive control, the samples were taken immediately after the drying time without applying any disinfectant solution. For the negative control, the liner was immersed in a non-contaminated milk and sampled after the drying time. A total of 64 samples were taken, 15 each with contamination by the different strains. Of these, 3 samples each were treated with the different disinfectants, as well as the sterile water. Further, 3 samples per pathogen were sampled as positive controls undisinfected. In addition, 4 negative controls, without contamination, were prepared.

### 2.2. Field Trial

Samples for the field trial were collected in July 2022 from a farm with 575 milking cows, after all animals had been milked and before the milking equipment was cleaned and disinfected. For this purpose, one of the liners of each cluster was disinfected with PAS at a concentration of 0.1% and one with PABS. The third liner was treated with sterile water, and the fourth was left untreated. The application of the disinfection solutions was carried out at an external temperature of 22 °C. The selection of the position of the different treatments was randomized by sticking a different colored tape to each liner of the milking cluster. Each color corresponded to the application of a different disinfectant. The selection was made randomly. To disinfect the liner, two spray shots of 0.85 mL were placed inside the liner held horizontally, using the same spray bottles as in the laboratory study (see Figure 1). During the exposure time of 30 s, the cluster was left hanging. A total of 160 swab samples were collected from 40 milking clusters using the same technique as in the laboratory experiment: 40 untreated samples, 40 samples with sterile water, 40 samples with PABS and 40 samples with PAS were taken. The samples were brought to the laboratory and refrigerated at 7 °C within two hours.

### 2.3. Sampling Technique

Samples were collected by a WDS method in accordance to DIN 10113-1: 1997-07 using cosmetic supply swabs with a bamboo handle and a tip made of bisphenol A (BPA)-free cotton (Outdoor Freakz GmbH, Zossen, Germany) that were heat-sterilized at 140 °C for 2 h to avoid contamination, as they were not sterile packaged (see Figure 1). The swab solution was a quarter-strength Ringer’s solution (Merck Kgaa, Darmstadt, Germany) to which 2.2% sodium thiosulfate (APPLICHEM GmbH, Darmstadt, Germany) and 0.2% catalase (Carl Roth GmbH & Co. KG, Karlsruhe, Germany) were added as disinhibitors. An amount of 3 mL of the solution was used as a swab medium. Two swabs were applied for one sample. The first swab was moistened with the solution by dipping it for 5 s, and excess liquid was squeezed out at the edge of the sample tube. After swabbing the surface, a second, dry swab was used on the same surface as the first swab. The sampling area was kept constant by taking all swab samples at a depth of 5 cm in a 360° rotation while the swab itself was rotated, passing the swab only once over the sampling area. The contact pressure applied to the cotton swab was sufficient to bend the wooden stick. After sampling, both swabs were placed in the same test tube. To avoid the contamination of the swab medium through the samplers, the bamboo handles of the swabs were broken off, when bringing them into the sample tube. The sampling was performed by 1 person in the laboratory trial and by 4 different persons in the field trial, with each person sampling 10 complete milking clusters.

### 2.4. Microbiological Analysis

Tubes with samples were vortexed for 60 s to transfer the pathogens adhering to the cotton tips of the swabs into the swab medium. Serial dilutions were plated on PCM Agar in duplicate (Carl Roth GmbH & Co. KG, Karlsruhe, Germany) in accordance with the §64 LFGB (German Food and Feed Code) method: L 00.00-54. For the lab trial, 1 mL and 0.1 mL of each sample were plated in duplicate. As the samples on the farm were assumed to be more contaminated, a further dilution step was made (10^−2^). The plates were counted after 72 h of incubation at 30 °C, using a colony counting method, considering all plates with growth between 10 and 300 colonies (see Figure 1). The weighted arithmetic mean in cfu/mL of all the evaluable dilution levels of a sample in accordance with §64 LFGB (German Food and Feed Code) method L 01.00-57 was converted to cfu per square centimeter (cfu/cm^2^) in adjustment to the swab area. For the calculation, the circumference of 7.85 cm of the inside of the liner at a depth of 5 cm was used and multiplied by the contact area of the swab tips to obtain a swab area of 6.28 cm^2^.

### 2.5. Statistical Analysis

The data obtained were collected in Microsoft Excel and analyzed using the SPSS 28.0 program, SPSS Inc. (Chicago, IL, USA). The outcome variable “cfu/cm^2^ of a pathogen” was transformed to approximate a normal distribution and tested with the Kolmogorov–Smirnov test. Since the data collected in the study did not show a normal distribution, they were normalized by adding 1 and applying the log10 transformation. Factors associated with the outcome variable were identified with an analysis of variance and post hoc analysis using the Bonferroni test to reveal significant differences between group means. Statistical significance was defined as *p* < 0.05.

### 2.6. Plasma Device and Production of PABS

**Figure 2:**
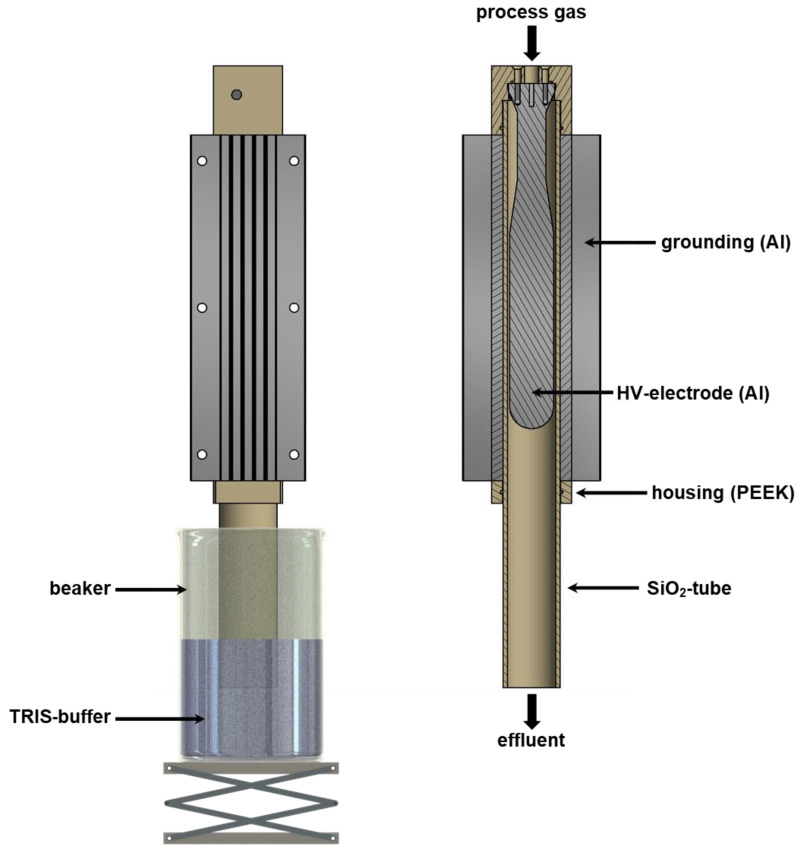
Scheme of the plasma device to generate PABS: (**left**), total view of the PAL device; (**right**), sectional view of the plasma source.

The PABS was produced using a plasma source developed by the faculty of engineering and health (HAWK, University of Applied Science and Arts, Göttingen, Germany) for the production of PABS based on the principle of a single-insulated dielectric barrier discharge (DBD). The complete setup for the generation of PABS consists of an outer radially symmetrical silica tube (total length: 300 mm, outer diameter: 46.7 mm, inner diameter: 43.3 mm) acting as insulator. A cooling unit consisting of an Al block with milled cooling fins, which also serves as the ground electrode (GND), surrounds the tube. Within the silica tube, an Al cylinder is centrally positioned acting as a high-voltage electrode (Figure 2). The Al cylinder has an outer diameter of 40 mm with a length of 400 mm. The geometry results in a discharge gap of approx. 1.7 mm, with a discharge length of approx. 200 mm (see Figure 2). The discharge gap is streamed with pressure air as it processes gas at a gas volume flow-rate of 50 L min^−1^. The quartz tube protrudes approx. 35 mm into a beaker filled with a 0.5 molar TRIS-buffer solution. The distance between the end of the discharge section and the water surface is approx. 190 mm, so that the plasma “exhaust” contacts water after approx. 0.4 s after exiting the plasma zone. The high-voltage power supply (Tantec HV-X20 Generator, transformer 13.5 kV, Denmark) provides sinusoidal waveform (U = 11.7 kV, peak-peak) with a repetition frequency of 17.2 kHz and an electrical power output of approx. 850 W. An amount of 0.25 L of TRIS-buffer (0.5 mol/L) out of TRIS(hydroxymethyl)aminomethan (TRIS, Trometamol, ≥99.8%, VWR International, Darmstadt, Germany) and TRIS-HCl (TRIS(hydroxymethyl)aminomethane hydrochlorid, ≥99.0%, VWR International, Darmstadt, Germany) was treated with the described plasma source for 40 min to obtain approx. 0.2 L PABS with a neutral pH value. According to Hoeben et al. [29], the determination of nitrate, nitrite and hydrogen peroxide is a quick way to conduct a basic characterization of PAL. A Reflectoquant (Merck KGaA, Darmstadt, Germany) was used to measure the concentrations of nitrate anions (NO_3_^−^; approx. 7700 mg/L) and nitrite anions (NO_2_^−^; approx. 1200 mg/L), as well as hydrogen peroxide (H_2_O_2_; approx. 2.7 mg/L) concentrations in the PABS. The PABS sample was transported at room temperature within 4 h after preparation to the Hanover University of Applied Sciences and Arts and stored at 7 °C for 12 h before performing the trials.

## 3. Results

### 3.1. Lab Trial

In the laboratory test, a total of 64 swab samples were collected from liners contaminated with the 4 different pathogens. The bacterial density in the milk was 2 × 10^4^ cfu/mL for *E. coli*, 1.5 × 10^4^ cfu/mL for *S. aureus*, 1 × 10^4^ cfu/mL for *Sc. uberis* and 2.4 × 10^4^ cfu/mL for *Sc. agalactiae*. Thus, for all different pathogens, the contamination on the liners was about 2.1 log_10_((cfu + 1)/cm^2^) (*E. coli*: 2.172 log_10_((cfu + 1)/cm^2^, *S. aureus*: 2.138 log_10_((cfu + 1)/cm^2^), *Sc. uberis*: 2.129 log_10_((cfu + 1)/cm^2^), *Sc. agalactiae*: 2.132 log_10_((cfu + 1)/cm^2^)).

The individual disinfectants achieved bacterial reductions of varying degrees for the different pathogens (Table 1). A reduction in pathogens also occurred when sterile water was used. Table 2 lists all multiple comparisons of the significant mean differences.

**Figure 3:**
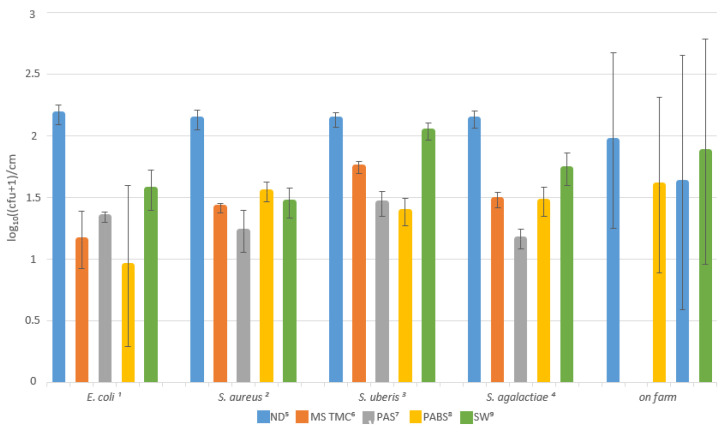
Comparison of surface microbial counts of liners not disinfected, and liners disinfected by the various methods with standard deviation in log_10_((cfu + 1)/cm^2^). ^1^ *Escherichia coli*. ^2^ *Staphylococcus aureus*. ^3^ *Streptococcus uberis*. ^4^ *Streptococcus agalactiae*. ^5^ Not disinfected. ^6^ MS TMC Flush. ^7^ Peracetic Acid Solution. ^8^ Plasma-Activated Buffered Solution. ^9^ Sterile water.

For all pathogens applied, a significant bacterial reduction was induced by the treatment of the liners with the disinfectants and the sterile water compared to the non-disinfected liners. Only for *Sc. uberis* did the sterile water show no significant effect (see Table 1). For *E. coli*, PABS reduced significantly more pathogens on the surface than sterile water. For *S. aureus* contamination, PAS achieved a significantly better reduction in bacteria than PABS (0.913 log_10_((cfu + 1)/cm^2^) vs. 0.595 log_10_((cfu + 1)/cm^2^)). Liners contaminated with *Sc. uberis* were best disinfected with PABS, achieving a reduction of 0.746 log_10_((cfu + 1)/cm^2^). All other disinfectants also achieved significant bacterial reductions, as well as significantly better reductions than when sterile water was used. Compared to PAS and PABS, the application of MS TMC Flush yielded insufficient pathogen reductions. On the liners contaminated with *Sc. agalactiae*, PAS achieved a reduction of 0.971 log_10_((cfu + 1)/cm^2^), a significantly better bacterial reduction than MS TMC Flush, PABS and sterile water. The bacterial reductions by each disinfection method are shown in Figure 3.

### 3.2. Field Trial

A total of 40 milking clusters were sampled and a mean contamination of the inner liner surfaces of 1.927 log_10_((cfu + 1)/cm^2^) was found. After disinfection with PAS, the surface bacterial count on the inner liner surfaces was 1.574 log_10_((cfu + 1)/cm^2^), after disinfection with PABS 1.774 log_10_((cfu + 1)/cm^2^). When the sterile water was applied, a surface bacterial count of 1.863 log_10_((cfu + 1)/cm^2^) was measured (Table 3).

When comparing the mean differences (Table 4), it is noticeable that neither the application of the disinfection solutions nor the sterile water resulted in a significant reduction in the bacterial density on the liners. A significant difference regarding sampler and udder quarter was also not found, when using tests of between-subjects effects. Figure 3 shows the reduction in the surface bacterial count by the individual disinfection solutions, also in comparison to the laboratory test.

## 4. Discussion

Intermediate disinfection is recommended as a measure to control the transmission of mastitis pathogens, especially on farms with problems with cow-associated pathogens, such as *S. aureus*, which are mainly transmitted during the milking process. Only by quantitatively detecting the surface microbial count on the liner surface it is possible to determine the level of the reduction in the bacteria on the liner surface by intermediate disinfection. A qualitative or semi-quantitative swab sample provides only unreliable information since no precise quantitative conclusion can be made about the surface bacterial count (DIN 10113-1:1997-07, Part 2). This study was a continuation of a previous experiment [38] that defined a modified WDS technique in accordance to DIN 10113-1: 1997-07, suitable for the quantitative swab sampling of milking liners. In the current study, by means of a laboratory trial and a field trial, the aim was to find out how different disinfectants act on certain bacterial species and how they reduce the bacterial count of the mixed flora on a teat cup liner. Even though there are already some studies dealing with bacterial reduction after intermediate disinfection, most of them lack standardized disinfection and sampling methods, as used in this study. For the laboratory trial, used liners made of NBR were deliberately chosen, because they favor pathogen deposition and thus transmission [40,41,42]. These were also installed on the farm where the field trial was performed. In the field trial, the samples were taken by four different samplers and not by one person as in the laboratory trial. All samplers swabbed the same number of clusters, so they each sampled the same number of differently disinfected and non-disinfected liners. There was no significant difference between the udder quarters or the samplers. Thus, the method used for sampling is sufficiently standardized to be used by different persons. To neutralize the bactericidal effect of disinfectant residues after sampling within the swab medium, a neutralizer additive consisting of sodium thiosulfate and catalase was added to the swab medium. This is important to determine the actual effect of the disinfectant on the surface of the liner and to avoid false-negative results [43,44,45,46]. Using this WDS technique, the effects of conventional disinfectants and PABS were compared within this study. To differentiate the extent to which the effect of rinsing by the liquid already on the liner surface alone leads to a reduction in the microorganisms on the surface, the liners were sprayed with sterile water in the study for comparison as is the case when the milking cluster is rinsed with cold water. Under controlled conditions in the laboratory, this reduction was notably lower than for most pathogen/disinfectant combinations. Under field conditions, the surface bacterial count was not significantly reduced by sterile water, nor by any of the tested disinfectants, so the risk of pathogen transmission is still present.

In the trials, a spray method was used to disinfect the liners. This method can be easily performed with minimal cost and material expenditure, which is why it is frequently used in Northern Germany for the intermediate disinfection of milking clusters. In addition, the disinfectant solution in the bottle is protected from contamination, unlike when using the immersion method, where protein fixation can occur due to milk residues on the milking cluster, which is minor but does occur when PAS is used [47]. Care must be taken to ensure that the insides of the liners are evenly wetted with the disinfection solution [22]. For the comparison of efficacy in the study, it was important to maintain both quantity and time exactly. Therefore, exactly 1.7 mL of the solutions was sprayed into the liners and the samples were taken after an exposure time of 30 s. This corresponds in application quantity and exposure time to the intermediate disinfection carried out in practice. However, the small amount of disinfectant and the short contact time are probably the reason for the low disinfection effects measured in the study [22,32,48].

The Robert Koch Institute’s definition of surface disinfection from 2022 calls for the destruction or inactivation of microorganisms to a level at which there is no longer any risk of infection. To achieve this, one would prefer a higher reduction in the bacteria on the inner surface of the liners in the laboratory trial, but it is especially important in the field trial. Although both PAS and MS TMC Flush reduce microorganisms on the liner surface, the average reductions in the microorganisms in the laboratory test were only 1 log for *E. coli* and 0.7 log, 0.7 log and 0.8 log for *S. aureus*, *Sc. uberis* and *Sc. agalactiae*, respectively. The different effects of the disinfectants on the bacteria probably arise from the different cell structures of Gram-positive and Gram-negative bacteria [49,50]. The higher resistance of *S. aureus* compared to *E. coli* regarding the oxidative effect of PAS was, for example, already presented by Kunigk and Almeida [21] and of PABS by Große Peclum et al. [31]. The best disinfection efficacy for *E. coli* and *Sc. uberis* in the laboratory study was obtained with PABS and for *S. aureus* and *Sc. agalactiae* with PAS. PABS achieved a reduction for *E. coli* by 1.3 log and for *Sc. uberis* by 0.8 log. PAS, however, achieved a reduction of 1.1 log in *S. aureus* and of 1 log in *Sc. agalactiae*.

Since the disinfection effect from the ready-to-use mix containing hydrogen peroxide, peracetic acid and acetic acid was worse than that of PAS and PABS in the laboratory trial, only the application of PAS and PABS on liners was compared with the rinsing effect of sterile water and non-disinfected liners in the field trial. The disinfectant efficacy trial under field conditions with liners contaminated with mixed flora resulted only in a 0.2 log reduction in bacteria and was thus much lower than in the laboratory. Zhang et al. [49] already described the effect of the lower efficacy of OH-based disinfectants with contamination by *E. coli* and *S. aureus* at the same time. Under field conditions, PAS and PABS did not lead to a significant reduction in the bacterial surface contamination of the milking liners, and there was also no significant difference between the two disinfection methods. An increase in the antimicrobial effect could probably be achieved by applying other PALs, since the pH value is kept neutral when using Buffered Solutions [32,51]. However, since PABS can be used for treatment on open wounds [31], it can be assumed that the sensitive teat skin is not stressed by PABS, making it particularly interesting for use on the liner. However, on the farm, none of the applied disinfectants showed a significant reduction in surface microbial counts. The low bacterial reductions contrast with the disinfection results obtained on the milking cluster by Skarbye et al. [36], who already achieved the bacterial elimination of *S. aureus* by rinsing with sterile water in a volume of 966 mL without the need to add PAS. So, in a further study on the milking cluster, it could be shown to what extent the disinfection effect of the various preparations’ changes with increased quantity and exposure time. Various laboratory studies already show that a higher microorganism reduction can be achieved with extended exposure time and that Gram-positive bacteria, such as *S. aureus,* then also show higher levels of destruction [20,24,31]. It can be assumed that conventional disinfectants, such as PAS and PABS, can then achieve good results, when used in the field.

Thus, PABS is called an environmentally friendly, non-toxic alternative to chemical disinfectants because the transfer of energy and chemical reactivity does not require any chemical additives [26] and it does not contain hazardous substances, unlike conventional disinfectants that must first be diluted before use. However, plasma activation requires a lot of electricity [30], which reduces the aspect of environmental friendliness. The disinfecting effect of PABS persists for 24 h when stored at 21 °C due to the long-lasting RONS [31,32], so PABS, like peracetic-acid-based disinfectants, must be prepared daily and applied after each milking [20]. However, the production of PABS is very power- and time-intensive and thus not yet possible on a widespread basis, but applications in further studies for disinfection purposes in the dairy industry are conceivable.

## 5. Conclusions

Both the conventional disinfectants based on peracetic acid and hydrogen peroxide and the PABS achieved a significant reduction in the surface microbial count on the liners in the laboratory test. In the field trial, the surface bacterial counts on the liners were reduced by PAS and PABS, but no significant difference was observed in liners that were not disinfected. This indicates that spray disinfection with the different solutions is able to reduce the contamination of milking liners, but a higher reduction in bacteria would be preferred for disinfection. In a subsequent study, both the amount of disinfectant solution applied and the exposure time of the disinfectants could be increased, to further reduce the microbial contamination of the liner and thus provide a more accurate conclusion about the effect of the different intermediate disinfectant solutions. Due to the lack of availability and the high costs of PABS, it is currently not an alternative to PAS in intermediate disinfection, despite the fact that it achieves comparable results.

## Figures and Tables

**Table 1:** Estimated marginal means for the pathogen density on the liners after disinfection in the laboratory study in log_10_((cfu + 1)/cm^2^).

Pathogen	Disinfectant	Mean (log_10_((cfu + 1)/cm^2^)) ^1^	95% CI ^2^ Lower Bound	95% CI ^2^ Upper Bound
*E. coli* ^3^	ND ^7^	2.172	1.992	2.412
	MS TMC ^8^	1.156 ^a,b^	0.907	1.406
	PAS ^9^	1.341 ^a^	1.092	1.591
	PABS ^10^	0.944 ^a,b,c^	0.695	1.194
	SW ^11^	1.559 ^a^	1.309	1.808
*S. aureus* ^4^	ND ^7^	2.138	1.998	2.277
	MS TMC ^8^	1.414 ^a^	1.274	1.553
	PAS ^9^	1.225 ^a,b,d^	1.086	1.365
	PABS ^10^	1.543 ^a^	1.403	1.683
	SW ^11^	1.456 ^a^	1.316	1.595
*Sc. uberis* ^5^	ND ^7^	2.129	2.032	2.236
	MS TMC ^8^	1.741 ^a,b^	1.635	1.848
	PAS ^9^	1.450 ^a,b,e^	1.343	1.556
	PABS ^10^	1.383 ^a,b,e^	1.277	1.489
	SW ^11^	2.036	1.930	2.142
*Sc. agalactiae* ^6^	ND ^7^	2.132	2.007	2.258
	MS TMC ^8^	1.479 ^a,b^	1.353	1.605
	PAS ^9^	1.162 ^a,b,d,e^	1.036	1.288
	PABS ^10^	1.466 ^a,b^	1.341	1.592
	SW ^11^	1.730 ^a^	1.604	1.856

^1^ Logarithmized colony-forming units per square centimeter. ^2^ Confidence Interval. ^3^ *Escherichia coli*. ^4^ *Staphylococcus aureus*. ^5^ *Streptococcus uberis*. *^6^ Streptococcus agalactiae*. ^7^ Not disinfected. ^8^ MS TMC Flush. ^9^ Peracetic Acid Solution. ^10^ Plasma-Activated Buffered Solution. ^11^ Sterile water. ^a^ Significant reduction in microorganisms. ^b^ Significant better reduction than SW. ^c^ Significant better reduction than PAS. ^d^ Significant better reduction than PABS. ^e^ Significant better reduction than MS TMC.

**Table 2:** All significant mean differences in surface bacterial counts in relation to the different disinfection methods for pathogens in the laboratory study in log_10_((cfu + 1)/cm^2^).

Pathogen	DM1 ^1^	DM2 ^1^	Mean Difference (log_10_((cfu + 1)/cm^2^)) ^2^ (DM1–DM2)	*p*-Value	95% CI ^3^ Lower Bound	95% CI ^3^ Upper Bound
*E. coli* ^4^	ND ^8^	MS TMC ^9^	1.015	<0.001	0.448	1.582
		PAS ^10^	0.830	0.004	0.264	1.397
		PABS ^11^	1.227	<0.001	0.661	1.794
		SW ^12^	0.613	0.031	0.459	1.180
	SW ^12^	PABS ^11^	0.615	0.003	0.262	0.967
*S. aureus* ^5^	ND ^8^	MS TMC ^9^	0.724	<0.001	0.407	1.041
		PAS ^10^	0.913	<0.001	0.595	1.229
		PABS ^11^	0.595	<0.001	0.277	0.912
		SW ^12^	0.682	<0.001	0.365	0.999
	PABS ^11^	PAS ^10^	0.318	0.049	0.001	0.635
*Sc. uberis* ^6^	ND ^8^	MS TMC ^9^	0.388	0.002	0.146	0.630
		PAS ^10^	0.680	<0.001	0.438	0.921
		PABS ^11^	0.746	<0.001	0.505	0.988
	MS TMC ^9^	PAS ^10^	0.292	0.015	0.050	0.533
		PABS ^11^	0.358	0.003	0.117	0.600
	SW ^12^	MS TMC ^9^	0.295	0.014	0.053	0.536
		PAS ^10^	0.587	<0.001	0.345	0.828
		PABS ^11^	0.653	<0.001	0.412	0.895
*Sc. agalactiae* ^7^	ND ^8^	MS TMC ^9^	0.654	<0.001	0.368	0.940
		PAS ^10^	0.971	<0.001	0.685	1.257
		PABS ^11^	0.666	<0.001	0.380	0.952
		SW ^12^	0.403	0.005	0.117	0.689
	MS TMC ^9^	PAS ^10^	0.317	0.027	0.308	0.603
	PABS ^11^	PAS ^10^	0.305	0.034	0.185	0.591
	SW ^12^	PAS ^10^	0.568	<0.001	0.282	0.854

^1^ Disinfection method. ^2^ Logarithmized colony-forming units per square centimeter. ^3^ Confidence Interval. ^4^ *Escherichia coli*. ^5^ *Staphylococcus aureus*. ^6^ *Streptococcus uberis*. ^7^ *Streptococcus agalactiae*. ^8^ Not disinfected. ^9^ MS TMC Flush. ^10^ Peracetic Acid Solution. ^11^ Plasma-Activated Buffered Solution. ^12^ Sterile water.

**Table 3:** Estimated marginal means for the pathogen density on the liners after disinfection in the field trial in log_10_((cfu + 1)/cm^2^).

Disinfectant	Mean (log_10_((cfu + 1)/cm^2^)) ^1^	95% CI ^2^ Lower Bound	95% CI ^2^ Upper Bound
ND ^3^	1.927	1.639	2.214
PAS ^4^	1.574	1.301	1.847
PABS ^5^	1.774	1.482	2.066
SW ^6^	1.863	1.575	2.150

^1^ Logarithmized colony-forming units per square centimeter. ^2^ Confidence Interval. ^3^ Not disinfected. ^4^ Peracetic Acid Solution. ^5^ Plasma-Activated Buffered Solution. ^6^ Sterile water.

**Table 4:** Mean differences in surface bacterial counts in relation to the different disinfection methods for pathogens in the field trial in log_10_((cfu + 1)/cm^2^).

DM1 ^1^	DM2 ^1^	Mean Difference (log_10_((cfu + 1)/cm^2^)) ^2^ (DM1–DM2)	*p*-Value	95 % CI ^3^ Lower Bound	95 % CI ^3^ Upper Bound
ND ^4^	PAS ^5^	0.353	0.483	−0.185	0.891
	PABS ^6^	0.153	1.000	−0.403	0.709
	SW ^7^	0.064	1.000	−0.487	0.615

^1^ Disinfection method. ^2^ Logarithmized colony-forming units per square centimeter. ^3^ Confidence Interval. ^4^ Not disinfected. ^5^ Peracetic Acid Solution. ^6^ Plasma-Activated Buffered Solution. ^7^ Sterile water.

## Data Availability

The data presented in this study are available on request from the corresponding author. The data are not publicly available due to privacy.
